# Blind loop: rare but important surgical complication

**DOI:** 10.1186/1471-2482-14-63

**Published:** 2014-08-31

**Authors:** John Michael DiBianco, Robert A Brenes, J Alexander Palesty

**Affiliations:** 1 The Stanley J. Dudrick Department of Surgery Saint Mary’s Hospital, Waterbury, CT, USA; 2 Ross University School of Medicine, Roseau, Commonwealth of Dominica, West Indies

**Keywords:** Enterocutaneous fistual, Fistula, Blind loop, Crohn’s disease, Surgical complications

## Abstract

**Background:**

Surgical complications worldwide are dreaded by both patients and physicians alike. They represent significant and serious morbidity and mortality, and contribute substantially to increased costs of healthcare.

**Case presentation:**

Our Case Report describes a 65yo Caucasian man with an extensive operative history for Crohn’s disease, including 4 laparotomies with small bowel resections to ameliorate small bowel obstructions. He presented with signs and symptoms of a chronic draining sinus, but was found to have a Blind Loop of bowel. This finding is believed to be the result of a surgical complication.

**Conclusion:**

While the Case Reports discusses this particular patient presentation, the paper defines, describes and offers treatment strategies for Enterocutaneous Fistulas (ECF). We offer aim to add Blind Loop to the differential diagnosis when presented with a patient with signs and symptoms of ECF.

## Background

Surgical complications invoke dread in both patients and physicians alike. They represent one of the most serious types of morbidity and at times may result in mortality. Negatively affecting the quality of life and wellbeing of our patients, these complications contribute substantially to increased costs of healthcare. One of the most feared surgical complications, due to the difficulty in treating and the profoundly negative impact on patient quality of life, is the enterocutaneous fistula (ECF); the incidence of which varies based on the type of operation, patient comorbidities and the expertise of the surgical team. Although the incidence of the ECF is relatively low in most reported series, when such complications arise, it is critical that their identification, cause and treatment be clearly elucidated so as to reduce the likelihood of similar complications in the future.

## Case presentation

**Figure 1:**
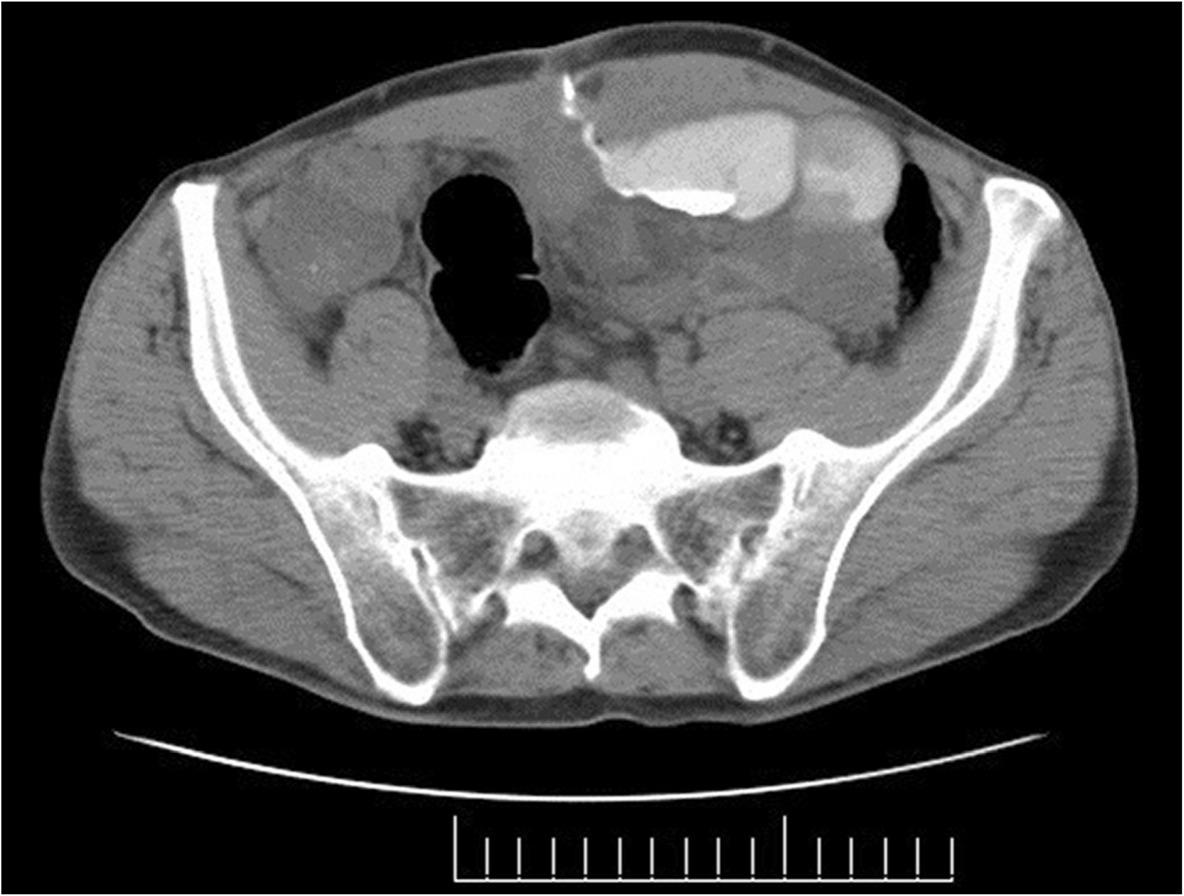
Patient CT fistulogram 2008.

A 65 year old male with an extensive operative history for Crohn’s disease, including 4 laparotomies with small bowel resections to ameliorate small bowel obstructions presented to us. His first surgery was performed in England in 1991, and the next 3 were performed in Peru, one in 1998 and two in 2007. Over the past few years he has been suffering from nutritional deficiencies and osteopenia associated with his resultant short bowel syndrome. He has had, in addition, intermittent drainage from his previous midline incision. The drainage was non-bilious, serosanguenous fluid that would egress approximately every 2–3 days. Several sutures were removed through the tract in the past but the wound still failed to heal. The drainage volume and quality did not change significantly over the past year. Although he did suffer from short bowel syndrome, he has had no change in his gastrointestinal symptomatology or body weight over the past year. On physical examination, his vital signs were within normal limits. His chest and abdominal examinations were unremarkable, except for an infra-umbilical surgical scar with, what appeared to be, a chronic sinus draining a minimal amount of serous fluid. A number of subcutaneous sutures were palpable, adjacent to the orifice of the sinus but no erythema or purulence was observed. A CT fistulogram done in 2008, 1 year after his most recent bowel resection, revealed a possible enterocutaneous fistula (Figure 1).

Given his history of inflammatory bowel disease, multiple prior surgical procedures and physical findings, the working diagnosis was a chronically draining abdominal wall sinus tract most likely secondary to a previously infected suture. The possibility of an enterocutaneous fistula was also entertained; however, his clinical picture, with minimal and intermittent drainage of non-succus fluid made the latter possibility less likely. The fistula noted on the CT fistulogram 5 years ago was believed to have closed given his presentation.

After explaining the risks, benefits and possible complications of surgical intervention, our patient elected to have a wound exploration with possible laparotomy under general anesthesia for the purpose resecting this sinus tract.

An elliptical incision was made around the suspected sinus tract and followed down to the fascia. A hemostat was placed in the lumen of the sinus tract which traveled below the fascia, necessitating entry into the abdomen. Sharp dissection along the previous midline incision revealed moderate to severely thickened abdominal wall adhesions, most likely a result of prior obliterative peritonitis. Extensive lysis of adhesions and mobilization of the small bowel took approximately 10 hours. Once the extensive adhesions were lysed and the bowel mobilized, the tract was traced to a 15 cm blind ended portion of small bowel, closed on one end with the fistulous tract emanating from the other. After running the bowel from the Ligament of Trietz to the peritoneal reflection multiple times revealing the rest of the bowel to be fully continuous, it was clear that the suspected sinus tract, was in fact, a fistula tract connected to a blind segment of small bowel which had its own mesenteric blood supply. A second surgeon was called into the OR to confirm the continuity of bowel and the intraoperative findings. Only after multiple confirmations of bowel continuity, was the tract and blind segment resected.

Postoperatively, our patient regained bowel function and promptly improved in overall health. At follow-up his wound was healing well without complications with resolution of his chronic draining sinus.

## Review

A fistula is an abnormal communication between 2 epithelialized surfaces, and an enterocutaneous fistula (ECF) is an abnormal communication between the bowel lumen and the skin, commonly draining intestinal contents to the environment [1]. They can be the result of inflammatory or infectious bowel disease, trauma and may occur following abdominal surgery. There is a wide variety of presentations, but the most common ECF presentation is in the post-operative febrile patient with a wound infection, abdominal distention and tenderness [1]. Presence of an ECF may be suspected when enteric contents are seen draining from the surgical wound, often beginning simultaneously with the drainage of purulent material but continuing whether or not associated infection resolves. ECFs are classified by the volume output per day: low output defined as < 200 mL/day, moderate output between 200 and 500 mL/day, and a high output ECF draining >500 mL/day [1]. According to this classification, the patient described in our case report had a low output ECF [1].

The differential diagnoses for low output enterocutaneous fistulas are: anastomotic leak, dehiscence, inadvertent enterotomy, inflammatory bowel disease, neoplasia, vascular insufficiency, radiation enteritis, mesenteric ischemia, diverticulitis, appendicitis, pancreatitis, tuberculosis, other intra-abdominal infections, abscesses, or malakoplakia [2]. Once an ECF is present, management options consist of: adequate nutrition, source control, negative pressure wound therapy, skin grafts, and ultimately surgical closure [3].

The goal of surgical management is to restore gastrointestinal tract continuity and complete separation of bowel lumen from abdominal wall tissues. Surgical management is reserved for patients whose management has failed for at least 5 weeks [3]. The surgical technique is variable and depends on the unique anatomy of each patient. It is often made more challenging because ECFs are associated with very dense adhesions which must be carefully dissected so as to avoid further damage and complications [4]. Once the appropriate incision is made and the adhesions lysed, the affected bowel should be resected, any enterotomies repaired, and the abdominal wall should be closed carefully [5].

## Discussion

With regards to our patient, the surprising intraoperative findings suggested that the remnant small bowel was inadvertently overlooked and left in the abdomen after one of his previous extended operations. The remnant piece of small bowel continued to secrete fluid, which, after building up enough pressure, escaped though the path of least resistance to the abdominal wall which was the sutured end of the small bowel and the site of a prior incision. This was supported by sutures found in proximity of the enterocutaneous fistula. The probability of this being an overlooked remnant loop was given additional credence after discussions with the patient revealed that the patient’s last operation was approximately 10 hours in duration and that the senior surgeon, due to his physical and mental exhaustion, turned over responsibility for completing the surgery, including closure of the abdomen, to a second surgeon, who may not have had as intimate an understanding of the anatomy, prior procedural details and what the senior surgeon had done and intended. During our procedure, the surgical director and chief resident were present throughout its entirety.

The rapidity of the patient’s recovery bolstered the diagnosis of enterocutaneous fistula secondary to a remnant loop of bowel with a chronic draining sinus. In retrospect, the diagnosis of a fistulized blind segment of bowel fits well with the clinical history and presentation. Since the segment of bowel was a blind loop, it would be expected to be of low output, with no source of succus or feculent drainage.

## Conclusions

This is an exceedingly rare type of enterocutaneous fistula with a paucity of literature available for review. It may be beneficial to add blind bowel loop to our differential when encountering a patient with a low output, non-bilious draining fistula, especially when it follows previous bowel surgery [5].

### Consent

Consent was obtained from both our patient and his wife during admission and then confirmed prior to discharge and once again at follow up where he provided us with a CD with his previous CT scan images. He was excited to allow us to share his story and clinical image in an attempt to educate and enlighten the medical community on a rare clinical diagnosis.

## Abbreviations

ECF: Enterocutaneous fistula; CT: Computerized tomography.

## Competing interests

The authors declare that they have no competing interests.

## Authors’ contributions

JAP is our patients surgeon who first met, examined and decided to treat our patient. JAP, RB and JD were all involved in the initial surgery, diagnosis and treatment of the patient. All the authors were responsible for researching, constructing and writing the first drafts of the manuscript. AP in addition, supervised and provided guidance for the structure of the paper and completed the final editing and revisions to all manuscript drafts. All authors read and approved the final manuscript.

## Pre-publication history

The pre-publication history for this paper can be accessed here:

http://www.biomedcentral.com/1471-2482/14/63/prepub

## References

[B1] Berry SM Fischer JE Classification and pathophysiology of enterocutaneous fistulas Surg Medical Clinics of North America 1996 76 5 1009 1018 10.1016/S0039-6109(05)70495-3 8841361

[B2] Tavakkolizadeh A Whang EE Ashley SW Zinner MJ Brunicardi F, Andersen DK, Billiar TR, Dunn DL, Hunter JG, Matthews JB, Pollock RE Small Intestine Schwartz’s Principles of Surgery, 9e 2010 New York, NY: McGraw-Hill

[B3] Schecter WP Hirshberg A Chang DS Harris HW Napolitano LM Wexner SD Dudrick SJ Enteric fistulas: principles of management J Am Coll Surg 2009 209 4 484 491 10.1016/j.jamcollsurg.2009.05.025 19801322

[B4] Shestak KC Edington HJ Johnson RR The separation of anatomic components technique for the reconstruction of massive midline abdominal wall defects: anatomy, surgical technique, applications, and limitations revisited Plast Reconstr Surg 2000 105 2 731 738 quiz 739 10.1097/00006534-200002000-00041 10697187

[B5] Hill GL Operative strategy in the treatment of enterocutaneous fistulas World J Surg 1983 7 4 495 501 10.1007/BF01655939 6414191

